# Joint Transmit Antenna Selection and Power Allocation for ISDF Relaying Mobile-to-Mobile Sensor Networks

**DOI:** 10.3390/s16020249

**Published:** 2016-02-19

**Authors:** Lingwei Xu, Hao Zhang, T. Aaron Gulliver

**Affiliations:** 1 College of Information Science and Engineering, Ocean University of China, Qingdao 266100, China; gaomilaojia2009@163.com (L.X.); zhanghao@ccompass.com.cn (H.Z.); 2 Department of Electrical and Computer Engineering, University of Victoria, Victoria, BC V8W 2Y2, Canada; agullive@ece.uvic.ca (T.A.G.)

**Keywords:** M2M communications, *N*-Nakagami fading channels, incremental-selective decode-and-forward, outage probability, transmit antenna selection, power allocation

## Abstract

The outage probability (OP) performance of multiple-relay incremental-selective decode-and-forward (ISDF) relaying mobile-to-mobile (M2M) sensor networks with transmit antenna selection (TAS) over *N*-Nakagami fading channels is investigated. Exact closed-form OP expressions for both optimal and suboptimal TAS schemes are derived. The power allocation problem is formulated to determine the optimal division of transmit power between the broadcast and relay phases. The OP performance under different conditions is evaluated via numerical simulation to verify the analysis. These results show that the optimal TAS scheme has better OP performance than the suboptimal scheme. Further, the power allocation parameter has a significant influence on the OP performance.

## 1. Introduction

To meet the increasing demands for high-data-rate services, mobile-to-mobile (M2M) communications has attracted significant interest from both industry and academia [1]. The M2M system architecture is described in [2]. In M2M communication systems, mobile users can directly communicate with each other without using a base station. This requires half the resources of traditional cellular communications, and thus improves the spectral efficiency and reduces the traffic load of the core network [3]. M2M communications can also be used to increase the data rate, reduce energy costs, reduce transmission delays, and extend the coverage area. Due to these advantages, M2M communications is an excellent choice for inter-vehicular communications, mobile sensor networks, and mobile heterogeneous networks [4]. M2M technologies have been proposed for home network applications [5]. In contrast to conventional fixed-to-mobile (F2M) cellular systems, both the transmitter and receiver in M2M systems can be in motion. Further, they are equipped with low elevation antennas. Thus, the widely employed Rayleigh, Rician, and Nakagami fading channels are not applicable to M2M communications systems [6]. Experimental results and theoretical analysis have shown that M2M channels can be described by cascaded fading channels [7]. The cascaded Rayleigh (also named as *N*-Rayleigh), fading channel was presented in [8]. The *N*-Rayleigh fading channel with *N* = 2, denoted the double-Rayleigh fading model, was considered in [9]. The *N*-Rayleigh fading channel was extended to the *N*-Nakagami fading channel in [10]. The *N*-Nakagami fading model with *N* = 2 is called the double-Nakagami fading model [11].

M2M communications may generate interference to existing cellular networks. To provide reliable cellular communications, the M2M transmit power and the distance between pairs of M2M users should be constrained. An M2M pair assisted by a relay can extend the coverage area with less transmit power. Therefore, relay-assisted M2M cooperative communications is an attractive solution to the interference problem. The pairwise error probability (PEP) of two relay-assisted vehicular scenarios using fixed-gain amplify-and-forward (FAF) relaying over double-Nakagami fading channels has been obtained [12]. An approximation for the average symbol error probability (SEP) has been derived for multiple-mobile-relay-based FAF relaying M2M cooperative networks over *N*-Nakagami fading channels [13]. Using the moment-generating function (MGF) approach, exact average SEP expressions for an AF M2M system over *N*-Nakagami fading channels have been derived [14].

Because of their low complexity, selective relaying (SR) and incremental relaying (IR) are widely employed in cooperative networks. Closed-form expressions for the error probability of incremental DF (IDF) and incremental AF (IAF) relaying over Rayleigh fading channels have been derived [15]. Further, an opportunistic IDF cooperation scheme employing orthogonal space-time block codes (OSTBC) over Rayleigh fading channels has been proposed [16]. In [17], closed-form OP expressions for IAF relaying M2M cooperative networks over *N*-Nakagami fading channels were derived. Exact average bit error probability (BEP) expressions for IDF relaying M2M cooperative networks over *N*-Nakagami fading channels were derived in [18].

Selective relaying (SR) cooperative networks are not efficient in terms of time and frequency resources. In IR cooperative networks, if the signal-to-noise ratio (SNR) of the link between the source and relay is low, messages may not be decoded correctly by the relay, which can cause error propagation. Considering these problems, Chen *et al.* [19] proposed a novel incremental-selective DF (ISDF) relaying scheme over Rayleigh fading channels which combines the IDF and SDF relaying protocols [19]. Exact closed-form OP expressions for ISDF relaying M2M cooperative networks over *N*-Nakagami fading channels were derived in [20].

Multiple-input-multiple-output (MIMO) transmission is a powerful technique which can be used to enhance the reliability and capacity of wireless systems. However, MIMO systems require multiple radio frequency chains, which increases the hardware complexity of the system. Transmit antenna selection (TAS) has been proposed as a practical way to reduce this complexity. In [21], the performance of optimal and suboptimal TAS schemes was investigated. Closed-form OP expressions for TAS MIMO networks over fading channels were derived in [22], and the energy efficiency was evaluated in [23].

Optimum power allocation is an important consideration in realizing the full potential of relay-assisted transmission. The resource allocation problem in both the uplink and downlink of two-tier networks comprising spectrum-sharing femtocells and macrocells has been investigated [24]. Further, the joint uplink subchannel and power allocation problem in cognitive small cells using cooperative Nash bargaining game theory was considered in [25]. A resource allocation scheme for orthogonal frequency division multiple access (OFDMA) based cognitive femtocells has been proposed [26], and secure resource allocation for OFDMA two-way relay wireless sensor networks was considered in [27].

However, to the best of our knowledge, the OP performance of ISDF relaying M2M sensor networks with TAS over *N*-Nakagami fading channels has not been investigated. Moreover, most results in the literature do not consider the power allocation. This is important, as it can have a significant effect on the OP performance. The main contributions of this paper are as follows:

1. Closed-form expressions for the probability density function (PDF) and cumulative density functions (CDF) of the SNR over *N*-Nakagami fading channels are presented. These are used to derive exact closed-form OP expressions for optimal and suboptimal TAS schemes. These expressions can be used to evaluate the performance of inter-vehicular networks, mobile wireless sensor networks, and mobile heterogeneous networks.
2. The power allocation problem is formulated to determine the optimum power distribution between the broadcast and relay phases.
3. The accuracy of the analytical results under different conditions is verified through numerical simulation. Results are given which show that the optimal TAS scheme has better OP performance than the suboptimal scheme. It is further shown that the power allocation parameter has a significant influence on the OP performance.
4. The OP expressions presented can be used to evaluate the performance of senor communication systems employed in inter-vehicular networks, mobile wireless sensor networks and mobile heterogeneous networks.

The rest of this paper is organized as follows. The multiple-mobile-relay-based M2M sensor network model is presented in Section 2. Section 3 provides exact closed-form OP expressions for the optimal TAS scheme. Exact closed-form OP expressions for the suboptimal TAS scheme are given in Section 4. In Section 5, the OP is optimized based on the power allocation parameter. Monte Carlo simulation results are presented in Section 6 to verify the analytical results. Finally, some concluding remarks are given in Section 7.

## 2. The System and Channel Model

### 2.1. System Model

**Figure 1:**
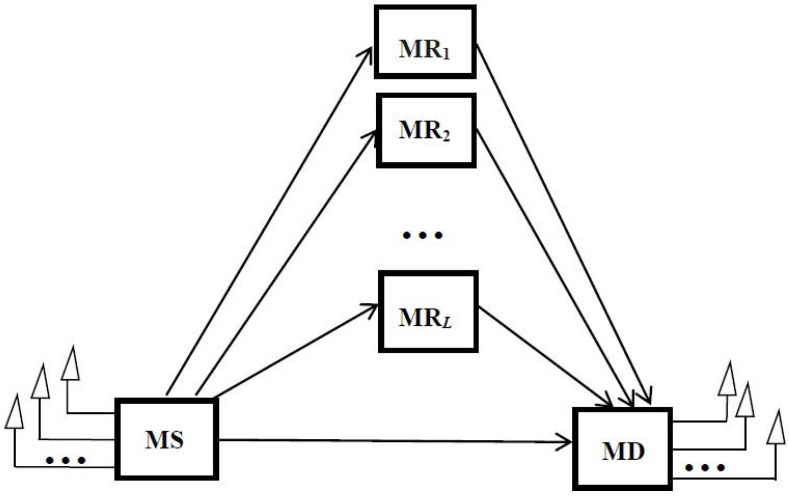
The system model.

The cooperation model consists of a single mobile source (MS) sensor, *L* mobile relay (MR) sensors, and a single mobile destination (MD) sensor, as shown in Figure 1. The nodes operate in half-duplex mode. The MS is equipped with *N_t_* antennas, the MD is equipped with *N_r_* antennas, and the MR is equipped with a single antenna.

It is assumed that the antennas at the MS and MD have the same distance to the relay nodes. Using the approach in [11], the relative gain of the MS to MD link is *G*_SD_ = 1, the relative gain of the MS to MR*_l_* link is *G*_SR*l*_ = (*d*_SD_/*d*_SR*l*_)*^v^*, and the relative gain of the MR*_l_* to MD link is *G*_RD*l*_ = (*d*_SD_/*d*_RD*l*_)*^v^*, where *v* is the path loss coefficient, and *d*_SD_, *d*_SR*l*_, and *d*_RD*l*_ are the distances of the MS to MD, MS to MR*_l_*, and MR*_l_* to MD links, respectively [28]. To indicate the location of MR*_l_* with respect to the MS and MD, the relative geometrical gain *μ_l_* = *G*_SR*l*_/*G*_RD*l*_ is defined. When MR*_l_* is closer to the MD, *μ_l_* is less than 1, and when MR*_l_* is closer to the MS, *μ_l_* is greater than 1. When MR*_l_* has the same distance to the MS and MD, *μ_l_* is 1 (0 dB).

Let MS*_i_* denote the *i*th transmit antenna at MS and MD*_j_* denote the *j*th receive antenna at MD. Further, let *h* = *h_k_*, *k*∈{SD*_ij_*, SR*_il_*, RD*_lj_*} represent the complex channel coefficients of the MS*_i_* to MD*_j_*, MS*_i_* to MR*_l_*, and MR*_l_* to MD*_j_* links, respectively. If the *i*th antenna at the MS is selected, during the first time slot the received signal *r*_SD*ij*_ at MD*_j_* is given by

(1) $$r_{\text{SD}ij}=\sqrt{KE}h_{\text{SD}ij}x+n_{\text{SD}ij}$$

and the received signal *r*_SR*il*_ at MR*_l_* by

(2) $$r_{\text{SR}il}=\sqrt{G_{\text{SR}il}KE}h_{\text{SR}il}x+n_{\text{SR}il}$$

where *x* denotes the transmitted symbol, and *n*_SR*il*_ and *n*_SD*ij*_ are additive white Gaussian noise (AWGN) with zero mean and variance *N*_0_/2. During the two time slots, *E* is the total energy used by the MS and MR, and *K* is the power allocation parameter.

During the second time slot, by comparing the instantaneous SNR *γ*_SD*ij*_ to a threshold *γ*_P_, only the best MR decides whether to be active.

If *γ*_SD*ij*_ > *γ*_P_, then MS*_i_* and the best MR will receive a ‘success’ message. MS*_i_* then transmits the next message, and the best MR remains silent. The corresponding received SNR at MD*_j_* is

(3) $$\gamma_{0ij}=\gamma_{\text{SD}ij}$$

where

(4) $$\gamma_{\text{SD}ij}=\frac{K{\left|h_{\text{SD}ij}\right|}^{2}E}{N_{0}}=K{\left|h_{\mathrm{SD}ij}\right|}^{2}\bar{\gamma }$$

If *γ*_SD*ij*_ < *γ*_P_, then MS*_i_* and the best MR will receive a ‘failure’ message. By comparing the instantaneous SNR *γ*_SR*i*_ to a threshold *γ*_T_, the best MR decides whether to decode and forward the signal to the MD*_j_*, where *γ*_SR*i*_ represents the SNR of the link between MS*_i_* and the best MR. The best MR is selected based on the following decision rule

(5) $$\gamma_{\text{SR}i}=\underset{1\leq l\leq L}{\max}(\gamma_{\text{SR}il})$$

where *γ*_SR*il*_ represents the SNR of the MS*_i_* to MR*_l_* link, and

(6) $$\gamma_{\text{SR}il}=\frac{KG_{\text{SR}il}{\left|h_{\text{SR}il}\right|}^{2}E}{N_{0}}=KG_{\text{SR}il}{\left|h_{\text{SR}il}\right|}^{2}\bar{\gamma }$$

If *γ*_Sr*i*_ < *γ*_T_, then MS*_i_* will transmit the next message, and the best MR will not be used for cooperation. The corresponding received SNR at MD*_j_* is

(7) $$\gamma_{1ij}=\gamma_{\text{SD}ij}$$

If *γ*_Sr*i*_ > *γ*_T_, then the best MR decodes and forwards the signal to MD*_j_*. The corresponding received signal at MD*_j_* is

(8) $$r_{\text{RD}j}=\sqrt{(1-K)G_{\text{RD}j}E}h_{\text{RD}j}x+n_{\text{RD}j}$$

where *n*_RD*j*_ is AWGN with zero mean and variance *N*_0_/2.

If MD*_j_* uses selection combining (SC), the received SNR is given by

(9) $$\gamma_{\text{SC}ij}=\max(\gamma_{\text{SD}ij},\gamma_{\text{RD}j})$$

where *γ*_RD*j*_ represents the SNR of the link between the best MR and MD*_j_*. Using SC at the MD, the received SNR is

(10) $$\gamma_{{\text{SC}}_{i}}=\underset{1\leq j\leq N_{r}}{\max}(\gamma_{ij})$$

where

(11) $$\gamma_{ij}=\{\begin{array}{c} \gamma_{0ij},\begin{array}{c} \gamma_{\text{SD}ij}>\gamma_{P} \end{array}\\ \gamma_{1ij},\begin{array}{c} \gamma_{\text{SD}ij}<\gamma_{P},\gamma_{\text{SR}i}<\gamma_{T} \end{array}\\ \gamma_{\text{SC}ij},\begin{array}{c} \gamma_{\text{SD}ij}<\gamma_{P},\gamma_{\text{SR}i}>\gamma_{T} \end{array} \end{array}$$

The optimal TAS scheme selects the transmit antenna *w* that maximizes the received SNR at the MD, namely

(12) $$w=\underset{1\leq i\leq N_{t}}{\max}(\gamma_{{\text{SC}}_{i}})=\underset{1\leq i\leq N_{t},1\leq j\leq N_{r}}{\max}(\gamma_{ij})$$

The suboptimal TAS scheme selects the transmit antenna *g* that maximizes the instantaneous SNR of the direct link MS*_i_* to MD*_j_*, namely

(13) $$g=\underset{1\leq i\leq N_{t},1\leq j\leq N_{r}}{\max}(\gamma_{\text{SD}ij})$$

### 2.2. System Model

The links in the system are subject to independent and identically distributed *N*-Nakagami fading, so that *h* follows the *N*-Nakagami distribution given by [10]

(14) $$h=\overset{N}{\underset{t=1}{\Pi }}a_{t}$$

where *N* is the number of cascaded components, and *a_t_* is a Nakagami distributed random variable with PDF

(15) $$f(a)=\frac{2m^{m}}{\Omega^{m}\Gamma (m)}a^{2m-1}\exp\left(-\frac{m}{\Omega }a^{2}\right)$$

Γ(·) is the Gamma function, *m* is the fading coefficient, and Ω is a scaling factor.

Using the approach in [10], the PDF of *h* is given by

(16) $$f(h)=\frac{2}{h\overset{N}{\underset{t=1}{\Pi }}\Gamma (m_{t})}G_{0,N}^{N,0}\left[h^{2}\overset{N}{\underset{t=1}{\Pi }}\frac{m_{t}}{\Omega_{t}}{|}_{m_{1},\ldots ,m_{N}}^{-}\right]$$

where *G*[·] is Meijer’s *G*-function.

Let *y* = |*h_k_*|^2^ represent the square of the amplitude of *h_k_*. The corresponding CDF and PDF of *y* are [10]

(17) $$F(y)=\frac{1}{\overset{N}{\underset{t=1}{\Pi }}\Gamma (m_{t})}G_{1,N+1}^{N,1}\left[y\overset{N}{\underset{t=1}{\Pi }}\frac{m_{t}}{\Omega_{t}}{|}_{m_{1},\ldots ,m_{N},0}^{1}\right]$$

(18) $$f(y)=\frac{1}{y\overset{N}{\underset{t=1}{\Pi }}\Gamma (m_{t})}G_{0,N}^{N,0}\left[y\overset{N}{\underset{t=1}{\Pi }}\frac{m_{t}}{\Omega_{t}}{|}_{m_{1},\ldots ,m_{N}}^{-}\right]$$

## 3. The OP of the Optimal TAS Scheme

The OP of the optimal TAS scheme can be expressed as

(19) $$F_{\text{optimal}}=\mathrm{Pr}(\underset{1\leq i\leq N_{t},1\leq j\leq N_{r}}{\max}(\gamma_{ij})<\gamma_{\text{th}})={\left(\mathrm{Pr}(\gamma_{ij}<\gamma_{\text{th}})\right)}^{N_{t}\times N_{r}}$$

### 3.1. γ_th_ > γ_P_

If *γ*_th_ > *γ*_P_, the OP of the optimal TAS scheme can be expressed as

(20) $$\begin{array}{ll} F_{\text{optimal}} & ={\left(\begin{array}{l} \mathrm{Pr}(\gamma_{p}<\gamma_{\text{SD}},\gamma_{0}<\gamma_{\text{th}})+\mathrm{Pr}(\gamma_{\text{SD}}<\gamma_{p},\gamma_{\text{SR}}<\gamma_{T},\gamma_{1}<\gamma_{\text{th}})\\ +\mathrm{Pr}(\gamma_{\text{SD}}<\gamma_{p},\gamma_{\text{SR}}>\gamma_{T},\gamma_{\text{SC}}<\gamma_{\text{th}}) \end{array}\right)}^{N_{t}\times N_{r}}\\ & ={\left(G_{1}+G_{2}+G_{3}\right)}^{N_{t}\times N_{r}} \end{array}$$

where *γ*_th_ is the threshold for correct detection at the MD. *G*_1_ is given by

(21) $$\begin{array}{l} G_{1}=\mathrm{Pr}(\gamma_{p}<\gamma_{\text{SD}},\gamma_{0}<\gamma_{\text{th}})=\mathrm{Pr}(\gamma_{p}<\gamma_{\text{SD}}<\gamma_{\text{th}})\\ =\frac{1}{\overset{N}{\underset{d=1}{\Pi }}\Gamma (m_{d})}\left(G_{1,N+1}^{N,1}\left[\frac{\gamma_{\text{th}}}{\bar{\gamma_{\text{SD}}}}\overset{N}{\underset{d=1}{\Pi }}\frac{m_{d}}{\Omega_{d}}{|}_{m_{1},\ldots ,m_{N},0}^{1}\right]-G_{1,N+1}^{N,1}\left[\frac{\gamma_{P}}{\bar{\gamma_{\text{SD}}}}\overset{N}{\underset{d=1}{\Pi }}\frac{m_{d}}{\Omega_{d}}{|}_{m_{1},\ldots ,m_{N},0}^{1}\right]\right) \end{array}$$

(22) $$\bar{\gamma_{\text{SD}}}=K\bar{\gamma }$$

*G*_2_ can be written as

(23) $$\begin{array}{l} G_{2}=\mathrm{Pr}(\gamma_{\text{SD}}<\gamma_{p},\gamma_{\text{SR}}<\gamma_{T},\gamma_{1}<\gamma_{\text{th}})\\ =\mathrm{Pr}(\gamma_{\text{SD}}<\gamma_{p},\gamma_{\text{SR}}<\gamma_{T})\\ =\frac{1}{\overset{N}{\underset{d=1}{\Pi }}\Gamma (m_{d})}G_{1,N+1}^{N,1}\left[\frac{\gamma_{P}}{\bar{\gamma_{\text{SD}}}}\overset{N}{\underset{d=1}{\Pi }}\frac{m_{d}}{\Omega_{d}}{|}_{m_{1},\ldots ,m_{N},0}^{1}\right]\times \\ {\left(\frac{1}{\overset{N}{\underset{t=1}{\Pi }}\Gamma (m_{t})}G_{1,N+1}^{N,1}\left[\frac{\gamma_{T}}{\bar{\gamma_{\text{SR}}}}\overset{N}{\underset{t=1}{\Pi }}\frac{m_{t}}{\Omega_{t}}{|}_{m_{1},\ldots ,m_{N},0}^{1}\right]\right)}^{L} \end{array}$$

(24) $$\bar{\gamma_{\text{SR}}}=KG_{\text{SR}}\bar{\gamma }$$

and *G*_3_ can be expressed as

(25) $$\begin{array}{l} G_{3}=\mathrm{Pr}(\gamma_{\text{SD}}<\gamma_{p},\gamma_{\text{SR}}>\gamma_{T},\gamma_{\text{SC}}<\gamma_{\text{th}})=\mathrm{Pr}(\gamma_{\text{SD}}<\gamma_{p},\gamma_{\text{SR}}>\gamma_{T},\gamma_{\text{RD}}<\gamma_{\text{th}})\\ =\frac{1}{\overset{N}{\underset{d=1}{\Pi }}\Gamma (m_{d})}G_{1,N+1}^{N,1}\left[\frac{\gamma_{P}}{\bar{\gamma_{\text{SD}}}}\overset{N}{\underset{d=1}{\Pi }}\frac{m_{d}}{\Omega_{d}}{|}_{m_{1},\ldots ,m_{N},0}^{1}\right]\times \frac{1}{\overset{N}{\underset{tt=1}{\Pi }}\Gamma (m_{tt})}G_{1,N+1}^{N,1}\left[\frac{\gamma_{\text{th}}}{\bar{\gamma_{\text{RD}}}}\overset{N}{\underset{tt=1}{\Pi }}\frac{m_{tt}}{\Omega_{tt}}{|}_{m_{1},\ldots ,m_{N},0}^{1}\right]\\ \times \left(1-{\left(\frac{1}{\overset{N}{\underset{t=1}{\Pi }}\Gamma (m_{t})}G_{1,N+1}^{N,1}\left[\frac{\gamma_{T}}{\bar{\gamma_{\text{SR}}}}\overset{N}{\underset{t=1}{\Pi }}\frac{m_{t}}{\Omega_{t}}{|}_{m_{1},\ldots ,m_{N},0}^{1}\right]\right)}^{L}\right) \end{array}$$

(26) $$\bar{\gamma_{\text{RD}}}=(1-K)G_{\text{RD}}\bar{\gamma }$$

### 3.2. γ_th_ < γ_P_

If *γ*_th_ < *γ*_P_, the OP of the optimal TAS scheme can be expressed as

(27) $$\begin{array}{ll} F_{\text{optimal}} & ={\left(\mathrm{Pr}(\gamma_{\text{SD}}<\gamma_{\text{th}},\gamma_{\text{SR}}<\gamma_{T},\gamma_{1}<\gamma_{\text{th}})+\mathrm{Pr}(\gamma_{\text{SD}}<\gamma_{\text{th}},\gamma_{\text{SR}}>\gamma_{T},\gamma_{\text{SC}}<\gamma_{\text{th}})\right)}^{N_{t}\times N_{r}}\\ & ={\left(G_{11}+G_{22}\right)}^{N_{t}\times N_{r}} \end{array}$$

where *G*_11_ can be written as

(28) $$\begin{array}{l} G_{11}=\mathrm{Pr}(\gamma_{\text{SD}}<\gamma_{\text{th}},\gamma_{\text{SR}}<\gamma_{T})\\ =\frac{1}{\overset{N}{\underset{d=1}{\Pi }}\Gamma (m_{d})}G_{1,N+1}^{N,1}\left[\frac{\gamma_{\text{th}}}{\bar{\gamma_{\text{SD}}}}\overset{N}{\underset{d=1}{\Pi }}\frac{m_{d}}{\Omega_{d}}{|}_{m_{1},\ldots ,m_{N},0}^{1}\right]\times {\left(\frac{1}{\overset{N}{\underset{t=1}{\Pi }}\Gamma (m_{t})}G_{1,N+1}^{N,1}\left[\frac{\gamma_{T}}{\bar{\gamma_{\text{SR}}}}\overset{N}{\underset{t=1}{\Pi }}\frac{m_{t}}{\Omega_{t}}{|}_{m_{1},\ldots ,m_{N},0}^{1}\right]\right)}^{L} \end{array}$$

and *G*_22_ is given by

(29) $$\begin{array}{l} G_{22}=\mathrm{Pr}(\gamma_{\text{SD}}<\gamma_{\text{th}},\gamma_{\text{SR}}>\gamma_{T},\gamma_{\text{RD}}<\gamma_{\text{th}})\\ =\frac{1}{\overset{N}{\underset{d=1}{\Pi }}\Gamma (m_{d})}G_{1,N+1}^{N,1}\left[\frac{\gamma_{\text{th}}}{\bar{\gamma_{\text{SD}}}}\overset{N}{\underset{d=1}{\Pi }}\frac{m_{d}}{\Omega_{d}}{|}_{m_{1},\ldots ,m_{N},0}^{1}\right]\times \frac{1}{\overset{N}{\underset{tt=1}{\Pi }}\Gamma (m_{tt})}G_{1,N+1}^{N,1}\left[\frac{\gamma_{\text{th}}}{\bar{\gamma_{\text{RD}}}}\overset{N}{\underset{tt=1}{\Pi }}\frac{m_{tt}}{\Omega_{tt}}{|}_{m_{1},\ldots ,m_{N},0}^{1}\right]\\ \times \left(1-{\left(\frac{1}{\overset{N}{\underset{t=1}{\Pi }}\Gamma (m_{t})}G_{1,N+1}^{N,1}\left[\frac{\gamma_{T}}{\bar{\gamma_{\text{SR}}}}\overset{N}{\underset{t=1}{\Pi }}\frac{m_{t}}{\Omega_{t}}{|}_{m_{1},\ldots ,m_{N},0}^{1}\right]\right)}^{L}\right) \end{array}$$

## 4. The OP of the Suboptimal TAS Scheme

### 4.1. γ_th_ > γ_P_

If *γ*_th_ > *γ*_P_, the OP of the suboptimal TAS scheme can be expressed as

(30) $$\begin{array}{l} F_{\text{suboptimal}}=\mathrm{Pr}(\gamma_{p}<\gamma_{\text{SD}g}<\gamma_{\text{th}})+\mathrm{Pr}(\gamma_{\text{SD}g}<\gamma_{p},\gamma_{\text{SR}}<\gamma_{T})+\mathrm{Pr}(\gamma_{\text{SD}g}<\gamma_{p},\gamma_{\text{SR}}>\gamma_{T},\gamma_{\text{RD}}<\gamma_{\text{th}})\\ \;\;\;\begin{array}{c} = \end{array}GG_{1}+GG_{2}+GG_{3} \end{array}$$

where

(31) $$\gamma_{\text{SD}g}=\underset{1\leq i\leq N_{t},1\leq j\leq N_{r}}{\max}(\gamma_{\text{SD}ij})$$

*GG*_1_ is given by

(32) $$\begin{array}{l} GG_{1}=\mathrm{Pr}(\gamma_{p}<\gamma_{\text{SD}g}<\gamma_{\text{th}})\\ ={\left(\frac{1}{\overset{N}{\underset{d=1}{\Pi }}\Gamma (m_{d})}G_{1,N+1}^{N,1}\left[\frac{\gamma_{\text{th}}}{\bar{\gamma_{\text{SD}}}}\overset{N}{\underset{d=1}{\Pi }}\frac{m_{d}}{\Omega_{d}}{|}_{m_{1},\ldots ,m_{N},0}^{1}\right]\right)}^{N_{t}\times N_{r}}-{\left(\frac{1}{\overset{N}{\underset{d=1}{\Pi }}\Gamma (m_{d})}G_{1,N+1}^{N,1}\left[\frac{\gamma_{P}}{\bar{\gamma_{\text{SD}}}}\overset{N}{\underset{d=1}{\Pi }}{|}_{m_{1},\ldots ,m_{N},0}^{1}\right]\right)}^{N_{t}\times N_{r}} \end{array}$$

*GG*_2_ can be written as

(33) $$\begin{array}{l} GG_{2}=\mathrm{Pr}(\gamma_{\text{SD}g}<\gamma_{p},\gamma_{\text{SR}}<\gamma_{T})\\ ={\left(\frac{1}{\overset{N}{\underset{d=1}{\Pi }}\Gamma (m_{d})}G_{1,N+1}^{N,1}\left[\frac{\gamma_{P}}{\bar{\gamma_{\text{SD}}}}\overset{N}{\underset{d=1}{\Pi }}\frac{m_{d}}{\Omega_{d}}{|}_{m_{1},\ldots ,m_{N},0}^{1}\right]\right)}^{N_{t}\times N_{r}}\times {\left(\frac{1}{\overset{N}{\underset{t=1}{\Pi }}\Gamma (m_{t})}G_{1,N+1}^{N,1}\left[\frac{\gamma_{T}}{\bar{\gamma_{\text{SR}}}}\overset{N}{\underset{t=1}{\Pi }}\frac{m_{t}}{\Omega_{t}}{|}_{m_{1},\ldots ,m_{N},0}^{1}\right]\right)}^{L} \end{array}$$

and *GG*_3_ can be expressed as

(34) $$\begin{array}{l} GG_{3}=\mathrm{Pr}(\gamma_{\text{SD}g}<\gamma_{p},\gamma_{\text{SR}}>\gamma_{T},\gamma_{\text{RD}}<\gamma_{\text{th}})\\ ={\left(\frac{1}{\overset{N}{\underset{d=1}{\Pi }}\Gamma (m_{d})}G_{1,N+1}^{N,1}\left[\frac{\gamma_{P}}{\bar{\gamma_{\text{SD}}}}\overset{N}{\underset{d=1}{\Pi }}\frac{m_{d}}{\Omega_{d}}{|}_{m_{1},\ldots ,m_{N},0}^{1}\right]\right)}^{N_{t}\times N_{r}}\times \frac{1}{\overset{N}{\underset{tt=1}{\Pi }}\Gamma (m_{tt})}G_{1,N+1}^{N,1}\left[\frac{\gamma_{\text{th}}}{\bar{\gamma_{\text{RD}}}}\overset{N}{\underset{tt=1}{\Pi }}\frac{m_{tt}}{\Omega_{tt}}{|}_{m_{1},\ldots ,m_{N},0}^{1}\right]\times \\ \left(1-{\left(\frac{1}{\overset{N}{\underset{t=1}{\Pi }}\Gamma (m_{t})}G_{1,N+1}^{N,1}\left[\frac{\gamma_{T}}{\bar{\gamma_{\text{SR}}}}\overset{N}{\underset{t=1}{\Pi }}\frac{m_{t}}{\Omega_{t}}{|}_{m_{1},\ldots ,m_{N},0}^{1}\right]\right)}^{L}\right) \end{array}$$

### 4.2. γ_th_ < γ_P_

If *γ*_th_ < *γ*_P_, the OP of the suboptimal TAS scheme can be expressed as

(35) $$\begin{array}{ll} F_{\text{suboptimal}} & =\mathrm{Pr}(\gamma_{\text{SD}g}<\gamma_{\text{th}},\gamma_{\text{SR}}<\gamma_{T})+\mathrm{Pr}(\gamma_{\text{SD}g}<\gamma_{\text{th}},\gamma_{\text{SR}}>\gamma_{T},\gamma_{\text{RD}}<\gamma_{\text{th}})\\ & =GG_{11}+GG_{22} \end{array}$$

where *GG*_11_ can be written as

(36) $$\begin{array}{l} GG_{11}=\mathrm{Pr}(\gamma_{\text{SD}g}<\gamma_{\text{th}},\gamma_{\text{SR}}<\gamma_{T})\\ ={\left(\frac{1}{\overset{N}{\underset{d=1}{\Pi }}\Gamma (m_{d})}G_{1,N+1}^{N,1}\left[\frac{\gamma_{\text{th}}}{\bar{\gamma_{\text{SD}}}}\overset{N}{\underset{d=1}{\Pi }}\frac{m_{d}}{\Omega_{d}}{|}_{m_{1},\ldots ,m_{N},0}^{1}\right]\right)}^{N_{t}\times N_{r}}\times {\left(\frac{1}{\overset{N}{\underset{t=1}{\Pi }}\Gamma (m_{t})}G_{1,N+1}^{N,1}\left[\frac{\gamma_{T}}{\bar{\gamma_{\text{SR}}}}\overset{N}{\underset{t=1}{\Pi }}\frac{m_{t}}{\Omega_{t}}{|}_{m_{1},\ldots ,m_{N},0}^{1}\right]\right)}^{L} \end{array}$$

and *GG*_22_ can be expressed as

(37) $$\begin{array}{l} GG_{22}=\mathrm{Pr}(\gamma_{\text{SD}g}<\gamma_{\text{th}},\gamma_{\text{SR}}>\gamma_{T},\gamma_{\text{RD}}<\gamma_{\text{th}})\\ ={\left(\frac{1}{\overset{N}{\underset{d=1}{\Pi }}\Gamma (m_{d})}G_{1,N+1}^{N,1}\left[\frac{\gamma_{\text{th}}}{\bar{\gamma_{\text{SD}}}}\overset{N}{\underset{d=1}{\Pi }}\frac{m_{d}}{\Omega_{d}}{|}_{m_{1},\ldots ,m_{N},0}^{1}\right]\right)}^{N_{t}\times N_{r}}\times \frac{1}{\overset{N}{\underset{tt=1}{\Pi }}\Gamma (m_{tt})}G_{1,N+1}^{N,1}\left[\frac{\gamma_{\text{th}}}{\bar{\gamma_{\text{RD}}}}\overset{N}{\underset{tt=1}{\Pi }}\frac{m_{tt}}{\Omega_{tt}}{|}_{m_{1},\ldots ,m_{N},0}^{1}\right]\times \\ \left(1-{\left(\frac{1}{\overset{N}{\underset{t=1}{\Pi }}\Gamma (m_{t})}G_{1,N+1}^{N,1}\left[\frac{\gamma_{T}}{\bar{\gamma_{\text{SR}}}}\overset{N}{\underset{t=1}{\Pi }}\frac{m_{t}}{\Omega_{t}}{|}_{m_{1},\ldots ,m_{N},0}^{1}\right]\right)}^{L}\right) \end{array}$$

## 5. Optimal Power Allocation

**Figure 2:**
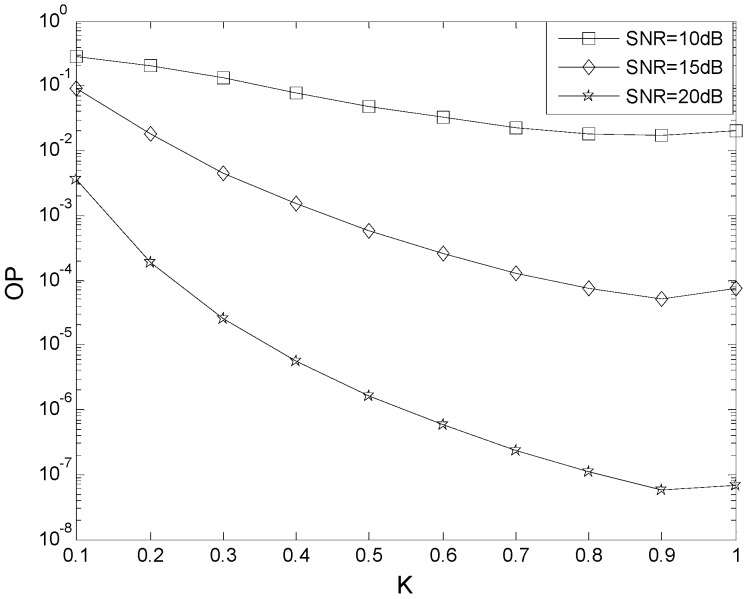
The effect of the power allocation parameter *K* on the OP performance.

Figure 2 presents the effect of the power allocation parameter *K* on the OP performance. The parameters are *N* = 2, *m* = 2, *μ* = 0 dB, *N_t_* = 2, *L* = 2, *N_r_* = 2, *γ*_th_ = 5 dB, *γ*_T_ = 2 dB, and *γ*_P_ = 2 dB. These results show that the OP performance improves as the SNR is increased. For example, when *K* = 0.7, the OP is 2.3 × 10^−2^ with SNR = 10 dB, 1.3 × 10^−4^ with SNR = 15 dB, and 2.4 × 10^−7^ with SNR = 20 dB. The optimum value of *K* is 0.86 with SNR = 10 dB, 0.92 with SNR = 15 dB, and 0.96 with SNR = 20 dB. This indicates that equal power allocation (EPA) is not the best scheme.

Unfortunately, it is very difficult to derive a closed-form expression for *K*. Because the OP expressions are very complex, numerical methods are used to solve the optimization problem. The optimum power allocation (OPA) values were obtained for given values of SNR and system parameters. Table 1 presents the optimum values of *K* for three values of relative geometrical gain *μ* = 5 dB, 0 dB, −5 dB. The other parameters are *N* = 2, *m* = 2, *N_t_* = 2, *L* = 2, *γ*_th_ = 5 dB, *γ*_T_ = 2 dB, and *γ*_P_ = 6 dB. For example, with a low SNR and *μ* = 5 dB, nearly all of the power should be used in the broadcast phase. As the SNR increases, the optimum value of *K* is reduced, so that at SNR = 20 dB only half of the power should be used in the broadcast phase.

**Figure 3:**
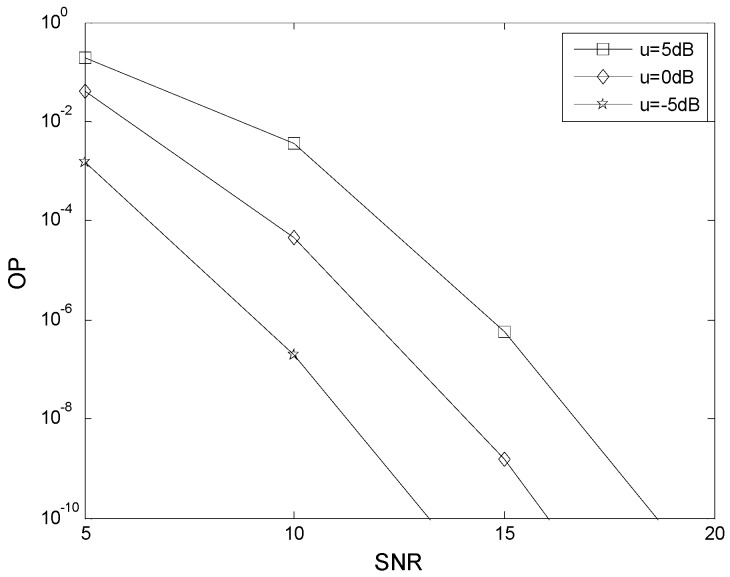
The effect of the relative geometrical gain *μ* on the OP performance.

Figure 3 presents the effect of the relative geometrical gain *μ* on the OP performance using the values of *K* given in Table 1. These results show that the OP performance improves as *μ* decreases. For example, when SNR = 10 dB, the OP is 3.6 × 10^−3^ for *μ* = 5 dB, 4.5 × 10^−5^ for *μ* = 0 dB, and 1.9 × 10^−7^ for *μ* = −5 dB. This indicates that the best location for the relay is near the destination. For fixed *μ*, an increase in the SNR reduces the OP, as expected.

## 6. Numerical Results

In this section, simulation results are presented to confirm the analysis given previously. The Monte Carlo simulations were done using MATLAB, and the analytical results were verified using MAPLE. The total energy is *E* = 1, the fading coefficient is *m* = 1, 2, 3, the number of cascaded components is *N* = 2, 3, 4, the number of mobile relays is *L* = 2, the number of receive antennas is *N_r_* = 2, the relative geometrical gain is *μ* = 0 dB, and the number of transmit antennas is *N_t_* = 1, 2, 3.

**Figure 4:**
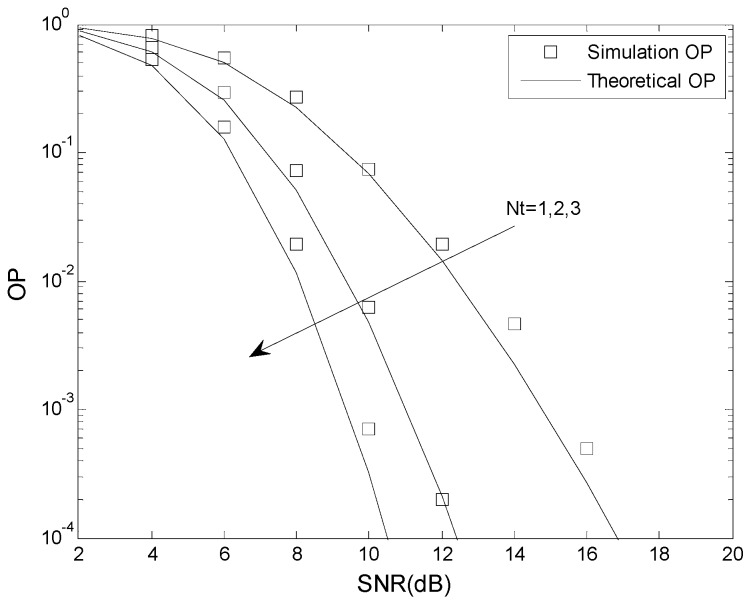
The OP performance of the optimal TAS scheme when *γ*_th_ < *γ*_P._

**Figure 5:**
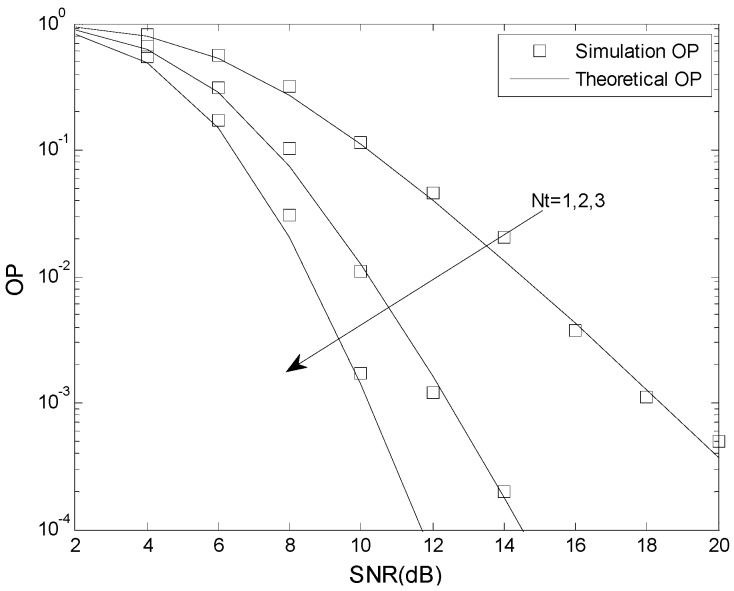
The OP performance of the optimal TAS scheme when *γ*_th_ > *γ*_P_.

Figure 4 and Figure 5 present the OP performance of the optimal TAS scheme with *γ*_th_ = 5 dB, *γ*_T_ = 2 dB and *γ*_P_ = 6 dB, and *γ*_th_ = 5 dB, *γ*_T_ = 2 dB and *γ*_P_ = 3 dB, respectively. The other parameters are *N* = 2, *m* = 2, *K* = 0.5, *N_t_* = 1, 2, 3, *L* = 2, *N_r_* = 2, and *μ* = 0 dB. This shows that the analytical results match the simulation results. The OP improves as the number of transmit antennas is increased. For example, when *γ*_th_ = 5 dB, *γ*_T_ = 2 dB, *γ*_P_ = 3 dB, and SNR = 10 dB, the OP is 1.1 × 10^−1^ when *N_t_* = 1, 1.3 × 10^−2^ when *N_t_* = 3, and 1.4 × 10^−3^ when *N_t_* = 3. For fixed *N_t_*, an increase in the SNR decreases the OP.

**Figure 6:**
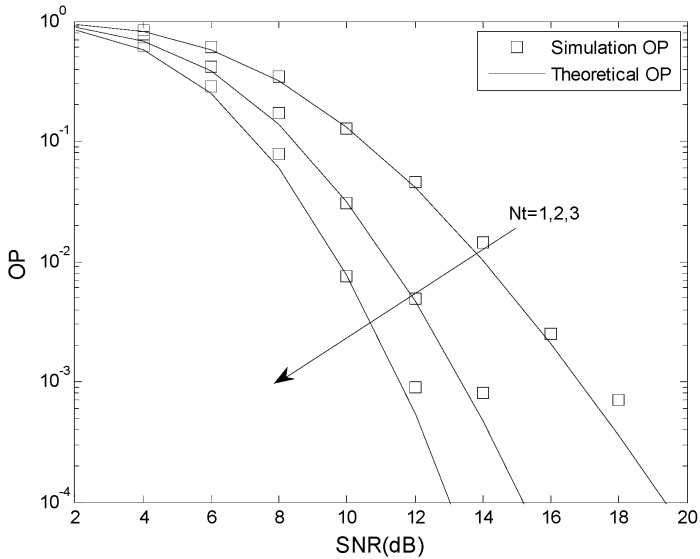
The OP performance of the suboptimal TAS scheme when *γ*_th_ < *γ*_P_.

**Figure 7:**
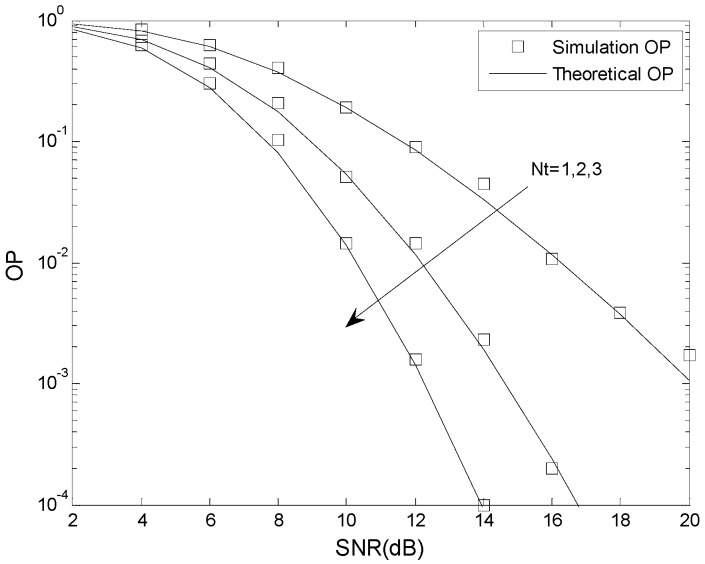
The OP performance of the suboptimal TAS scheme when *γ*_th_ > *γ*_P_.

Figure 6 and Figure 7 present the OP performance of the suboptimal TAS scheme with *γ*_th_ = 5 dB, *γ*_T_ = 2 dB and *γ*_P_ = 6 dB, and *γ*_th_ = 5 dB, *γ*_T_ = 2 dB and *γ*_P_ = 3 dB, respectively. The other parameters are *N* = 2, *m* = 2, *K* = 0.5, *N_t_* = 1, 2, 3, *L* = 2, *N_r_* = 2, and *μ* = 0 dB. This also shows that the analytical results match the simulation results. As expected, the OP improves as the number of transmit antennas is increased. For example, when *γ*_th_ = 5 dB, *γ*_T_ = 2 dB, *γ*_P_ = 6 dB, SNR = 12 dB, and *N_t_* = 1, the OP is 4.1 × 10^−2^ when *N_t_* = 1, 4.7 × 10^−3^ when *N_t_* = 2, and 5.3 × 10^−4^ when *N_t_* = 3, For fixed *N_t_*, an increase in the SNR decreases the OP, as expected.

**Figure 8:**
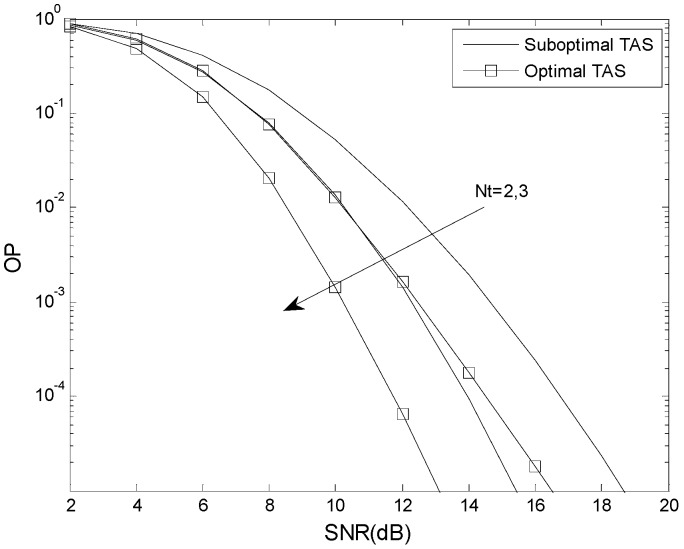
The OP performance of the optimal and suboptimal TAS schemes for different numbers of antennas *N_t_*.

Figure 8 compares the OP performance of the optimal and suboptimal TAS schemes for different numbers of antennas *N_t_*. The parameters are *N* = 2, *m* = 2, *K* = 0.5, *μ* = 0 dB, *N_t_* = 2, 3, *L* = 2, *N_r_* = 2, *γ*_th_ = 5 dB, *γ*_T_ = 2 dB, and *γ*_P_ = 3 dB. In all cases, for a given value of *N_t_* the optimal TAS scheme has better OP performance. As predicted by the analysis, the performance gap between the two TAS schemes decreases as *N_t_* is increased. When the SNR is low, the OP performance gap between the optimal TAS scheme with *N_t_* = 2 and the suboptimal TAS scheme with *N_t_* = 3 is negligible. As the SNR increases, the OP performance gap also increases.

## 7. Conclusions

In this paper, exact closed-form OP expressions were derived for ISDF relaying M2M networks with TAS over *N*-Nakagami fading channels. Performance results were presented which show that the optimal TAS scheme has better OP performance than the suboptimal scheme. It was also shown that the power allocation parameter *K* can have a significant effect on the OP performance. The given expressions can be used to evaluate the OP performance of inter-vehicular networks, mobile wireless sensor networks, and mobile heterogeneous networks.

## Figures and Tables

**Table 1:** OPA parameters *K*.

SNR (dB)	*μ* = 5 dB	*μ* = 0 dB	*μ* = −5 dB
5	0.99	0.41	0.51
10	0.51	0.41	0.47
15	0.50	0.44	0.45
20	0.50	0.47	0.46
